# Mania a Potu

**Published:** 1829

**Authors:** 


### II. Analytical.

29 *Mania apotu.*—Having considered this malady at some length, in reference to jurisprudence, we are tempted to resume it as a subject of practical medicine. The best, and only monographic treatise on this disease, is that of the learned and accurate Dr. Coates, one of the able Editors of the North American Medical and Surgical Journal, and contained in the 7th and 8th numbers of that work. It is not our design to analyse these interesting papers, which contain a synopsis of nearly all that was known on the subject, at the time they were written, but to lay before our readers the conclusions of the author, as summed at the end of the second paper. They are as follow:—

1. The disease is a delirium, and not a mania; and this distinction should be attended to, both for medical and legal reasons.

2. It consists in a heightened activity of the sensorium; and this appears to arise from the generation, in that organ, of an unusual vital power, which is not, as in common, exhausted by the narcotic poisons habitually used. This is not considered as an hypothesis, but the expression of a fact existing in nature.

3. The delirium may be combined with other diseases and injuries situated in many different parts of the body.

4. When violent, it obscures and renders imperceptible most of the symptoms of the co-existing disease.

5. It is doubtless necessarily accompanied, as all vital excitements are, with an unusual amount of the circulation of the blood in the organ affected; and is, from this cause, sensibly influenced by cups, blisters and emetics. It is not so far checked by the use of emetics as to render these advisable as a leading means of cure. It is not sufficiently under the control of the general circulation to be cured by venesection, or to be sensibly relieved by it without such an exhaustion as is highly dangerous to life.

6. It is entirely and absolutely under the controul of opium; although the fevers and other diseases which are liable to accompany it may be by no means so.

7. It admits of very large doses of opium, which are not productive, either at the time or subsequently, of any injurious consequences, provided they are not repeated after a tendency to sleep is evinced.

8. The patient must *sleep or die.* There is no alternative. Yet the physician should personally watch the effect of very large doses of opium.

9. There is no distinction of stages which need occasion a moment's delay in resorting to opium.

10. Purgatives are of no use in this delirium; but it is necssary to prevent costiveness subsequently to the administration of opium. Purgatives may be necessary for diseases which exist at the same time; but when this is the case, they are, in general, most advantageously postponed till after sleep has been obtained.

11. Gentle stimulants are frequently useful during the convalescence; but these should not resemble ardent spirits, and an excellent and sufficient one is capsicum. Nor should any ardent spirits, unless indicated by peculiar circumstances, be given during the paroxysm.

In the Medico-Chirurgical Review, for July, 1826, we find a notice of a paper on delirium tremens, by Mr. G. Playfair, published in the Transactions of the Calcutta Society, which as it goes to illustrate the phenomena and treatment of this disease in the East Indies, we shall extract.

In some instances, say the Reviewers, the restlessness continues, with particular wildness in the eye, and a dislike or a want of power to remain quiet. These symptoms gradually increase—he cannot lie down—or, if he does, he starts up as if likely to be suffocated. He talks in a strange manner, yet with the appearance of being perfectly in his senses; complains of the other patients making faces at him, and entering into conspiracies against him, &c. This is accompanied, of course, by want of sleep. It now becomes necessary to separate the patient from others to prevent mischief—and restraint itself is soon indispensable. Our author's experience does not coincide with those who believe that the disease only takes place in advanced life; on the contrary, he has found it almost invariably affect the young, the religious, the robust, the plethoric, and especially those who have been suddenly debilitated by previous illness. The mode of treatment in this case is to procure sleep, as this has always relieved the complaint. With this view, a full anodyne draught was exhibited at bed-time, and sometimes repeated at midnight, if not efficient by that time. When symptoms of mental derangement took place, he commenced with, perhaps, 100 drops of tincture of hyoscyamus, and 30 of laudanum, repeating 20 of the former, and ten of the latter, every two hours, as regularly as possible till sleep was procured. He has succeeded in twelve hours, but it has required 24, and even 36 hours before irritation could be allayed or sleep procured. He has been obliged to suspend the use of opium, till the bowels were well evacuated, but he generally continued the use of henbane. On several occasions he has been obliged to have recourse to the lancet, alarmed by the fulness of the pulse, inflamed appearance of the eyes, and obstinate constipation of the bowels. After the bleeding, opiates had a better effect. Tremors were not always present—the patient seldom complained of headache—the pulse was generally regular, and rather full—yet he has seen it feeble, and nearly imperceptible, the body being cold, and the sweat clammy. In these cases he has resorted to wine and other stimulants. Patients of this kind are almost always bathed in sweat—the tongue usually clean and moist, with great thirst. No case, in our author's practice, proved fatal; and in this, we think, he was very fortunate, for it is a dangerous disease.

In the above Review we observe the report of a discussion on Delirium Tremens, at a meeting of the Westminster Medical Society, in which the Editor, Dr. Johnston, Dr. Stewart, Dr. Copeland, Dr. Ayre and several other distinguished physicians of the British Metropolis, stated the results of their experience. These are altogether in favor of the narcotico-stimulant treatment. In the opinion of all who spoke, opium is the remedy on which the chief reliance should be placed; but most of them allowed the simultaneous exhibition of the same stimulating potation to which the patient had been accustomed.

We also find in the same Journal for August, 1828, an account of a memoir on *le Delire Tremblant*, by Dr. Leveille, which may be regarded as offering a view of the practice in France at the present time. When uncomplicated with arachnitis or gastritis, opium, according to the experience of Dr. L. is quite a specific for this malady, at whatever period it is administered. He gives the medicine in *large* doses. Our author is of opinion that the disease is occasioned by the absorption of alcohol and the application of its molecules directly to the cerebral matter.

In the third edition of his Practice of Physic, (1828) Dr. Gregory sums up the treatment of Delirium Tremens in the following paragraph:—

It is universally admitted that this complaint does not admit of depletion by blood-letting. Much mischief indeed has followed its employment. Leeches however are occasionally useful, and sometimes in its early stage indispensable. The principal aim of the physician should be to calm and support the nervous system, and if possible to procure sleep. Opium answers all these indications, and must be given in full and frequently repeated doses. Where the complaint can be traced distinctly to the excessive use of ardent spirits, the accustomed stimulus must not be too rapidly withdrawn. Wine or brandy in moderate quantities, should be administered.—Æther, ammonia, comphor, and hyosciamus, have also been found beneficial. It is unnecessary to add, that moderate purging should also be directed, for in so disordered state of the nervous system, the secretions can scarcely fail to be greatly vitiated.

Such are the principal recent notices, with which we have met, relative to the treatment of Delirium Tremens. Our readers will observe, that they all bear witness to the good effects of the opium practice, rather than the emetic. To the efficacy of this practice, we beg leave to add our own humble testimony, having, on the whole, accomplished more with it than any other. We have several times, however, seen excellent effects from the administration of emetics; and when the patient is neither old nor broken down in constitution, we prefer to commence the treatment with a vomit. This will, of itself, sometimes resolve the paroxysm, at other times it effects a useful preparation of the system, and gives greater efficacy to the opium. Indeed, the influence of emetics in augmenting the susceptibility of the stomach, is so great, that we are inclined to think they might be employed in most cases of Delirium Tremens with advantage. They would doubtless facilitate the narcotic effects which we seek to produce, and in many cases do away the necessity of administering drachms of opium. In shattered constitutions, laudanum may be advantageously combined with the emetic.

Of blood-letting we have but little to say. As a general rule, it is not necessary—perhaps not even safe, unless promptly followed by the administration of opium. But the sanguiferous system may be plethoric, or some organ or tissue may be inflamed, when venesection will undoubtedly be necessary. Of purging we would speak in similar language.

